# Effect of Non-Anticoagulant N-Desulfated Heparin on Basic Fibroblast Growth Factor Expression, Angiogenesis, and Metastasis of Gastric Carcinoma In Vitro and In Vivo

**DOI:** 10.1155/2012/752940

**Published:** 2012-07-25

**Authors:** Jin-Lian Chen, Jing Fan, Ming-Xiang Chen, Ying Dong, Jian-Zhong Gu

**Affiliations:** ^1^Department of Gastroenterology, Shanghai East Hospital, Tongji University School of Medicine, Shanghai 200120, China; ^2^Department of Gastroenterology, Shanghai Sixth People's Hospital, Shanghai Jiaotong University, Shanghai 200233, China; ^3^Shanghai Laboratory Animal Center, Chinese Academy of Sciences, Shanghai 200031, China

## Abstract

*Objective*. The present study was performed to investigate the effect of N-desulfated heparin on basic fibroblast growth factor (bFGF) expression, tumor angiogenesis and metastasis of gastric carcinoma. *Methods*. Human gastric cancer SGC-7901 tissues were orthotopically implanted into the stomach of NOD SCID mice. Twenty mice were randomly divided into two groups which received either intravenous injection of 0.9% NaCl solution (normal saline group) or 10 mg/kg N-desulfated heparin (N-desulfated heparin group) twice weekly for three weeks. In vitro, human gastric carcinoma SGC-7901 cells were treated with N-desulfated heparin in different concentration (0.1 mg/mL, 1 mg/mL, N-desulfated heparin group), and treated with medium (control group). *Results*. In vivo, the tumor metastasis rates were 9/10 in normal saline group and 2/10 in N-desulfated heparin group (*P* < 0.05). The intratumoral microvessel density was higher in normal saline group than in N-desulfated heparin group (*P* < 0.05). bFGF expression in gastric tissue was inhibited by N-desulfated heparin (*P* < 0.05). There was no bleeding in N-desulfated heparin group. In vitro, N-desulfated heparin inhibited significantly bFGF protein and mRNA expression of gastric carcinoma cells (*P* < 0.05). *Conclusions*. N-desulfated heparin can inhibit the metastasis of gastric cancer through inhibiting tumor bFGF expression and tumor angiogenesis with no obvious anticoagulant activity.

## 1. Introduction

Gastric cancer is the common alimentary tract cancer in China in terms of incidence. It is one of the malignancies that do serious harm to people's health with a high mortality and are short of effective therapeutic methods. Recent studies have showed that angiogenesis plays a crucial role in tumor growth and metastasis. Angiogenesis, which is the process by which new blood vessels develop from preexisting vessels, is governed by a very complex network of opposing signals that, under normal physiological conditions, are elicited by various highly regulated angiogenesis stimulators and inhibitors [1]. Angiogenesis is essential for tumor growth beyond a few millimeters in diameter because of the tumor's requirement for a network of blood vessels to deliver oxygen and nutrients and to remove waste products of metabolism. During tumor-associated angiogenesis, the balance of angiogenesis stimulators and inhibitors is tipped in favor of angiogenesis by hypoxia-inducible factor-1 gene expression [2]. Inhibition of angiogenesis can control tumor metastasis and improve the prognosis [3–6]. Vascular endothelial growth factor (VEGF) and fibroblast growth factor-2 (FGF-2) are the main factors promoting angiogenesis [7, 8]. Even though VEGF is a primary mediator of angiogenic responses, bFGF is more potent than VEGF for stimulating the vascular endothelial mitogenesis. Anti-VEGF therapy is effective in inhibiting angiogenesis and metastasis of tumor [9, 10]. Heparin, a highly sulfated proteoglycan, has been extensively used as an anticoagulant drug for a long time. Aside from its anticoagulant action, heparin binds to various growth factors, cytokines, and extracellular proteins and consequently is able to affect migration of cancer cells and angiogenesis in tumors. The potential anticancer activity of heparins is supported by data from in vitro and experimental studies [11]. Stevenson et al. [12] has reported that heparin primarily reduces metastatic disease by inhibiting P- and L-selectin interactions. However, clinical use of heparin in treatment of tumor is limited by its strong anticoagulant activity, which may cause severe bleeding complications. Chemically modified heparin shows a significantly reduced anticoagulant activity and enhanced ability to interact with FGF, VEGF, and hepatocyte growth factor, which are known to stimulate angiogenesis [13]. In this study, we investigated the effect of N-desulfated heparin on bFGF expression, angiogenesis, and tumor metastasis in vitro and in vivo.

## 2. Materials and Methods

### 2.1. Materials

Goat anti-human CD34 antibody and goat anti-human bFGF antibody were obtained from Santa Cruz Biotechnical Company. bFGF probe for real-time PCR was provided by Daan Gene Company of Zhongshan University. Human gastric adenocarcinoma SGC-7901 cell line was obtained from the Cell Biology Institute of Chinese Academy of Sciences, Shanghai.

### 2.2. Gastric Cancer Cell Cultivation

Human gastric cancer SGC-7901 cells were maintained in RPMI-1640 supplemented with 10% fetal bovine serum (FBS), 37°C in a humidified atmosphere containing 5% CO_2_. Human gastric cancer SGC-7901 cells were suspended at a concentration of 3 × 10^6^/10 mL. The different concentrations (0.1 mg/mL, 1 mg/mL) of N-desulfated heparin and medium were added to the cells. Cells were harvested after 12 h and 24 h. Cells were washed once with PBS and scraped into a wash buffer. The cells were washed in the buffer, homogenized in 150 *μ*L cell lysis buffer, and incubated on ice for 30 min. The supernatants were recovered and snap-frozen in liquid nitrogen and stored at −80°C.

### 2.3. Enzyme-Linked Immunosorbent Assay

A 96-well microwell plate (Nunc, Kamstrup, Denmark) was coated with 100 *μ*L of a 1 : 4000 dilution of purified IgY-anti bFGF in sodium bicarbonate buffer (pH 9.6) and incubated at 4°C overnight. The plate was washed twice with PBS containing 0.05% Tween 20 (PBST), and 100 *μ*L of recombinant bFGF-p24 was added, diluted 1 : 100 in 5 g/L pluripeptone, 3 g/L meat extract containing 0.05% Tween 20 (PMET). Incubation was performed at 37°C for 120 min. The microwell plate was washed four times with PBST and incubated at 37°C for 60 min with 100 *μ*L monoclonal antibody against p24 (VMRD), diluted 1 : 2000 in PMET. The plate was washed four times with PBST and incubated 60 min at 37°C with 100 *μ*L of anti-mouse-peroxidase diluted 1 : 4000 in PBST. The plate was washed again with PBS and detected with 90 *μ*L of tetramethylbenzidine (TMB) for 5~10 min at 37°C. The reaction was stopped by addition of 30 *μ*L of 2 mol/L H_2_SO4. Optical density value (OD) was measured at a wavelength of 492 nm. Each assay was performed three times, and the average results were calculated, using Ascent software for Multiskan reader.

### 2.4. Animal Model

Male NOD severe combined immune deficiency (SCID) mice were obtained from Shanghai Experimental Animal Center of Chinese Academy of Sciences. Animal experimental procedures were performed according to the relative ethical regulations for the care and use of laboratory animals of our university. Animals used were 6 weeks old and weighed 20–25 g. Human gastric cancer SGC-7901 (Shanghai Cancer Institute), a poorly-differentiated adenocarcinoma line, was originally derived from a primary tumor and maintained by passage in the subcutis of nude mice. Animal models were made using orthotopic implantation of histologically intact tissue of human gastric carcinoma [14]. Tumors were resected aseptically. Necrotic tissues were cut, and the remaining healthy tumor tissues were scissor minced into pieces (about 5 mm × 7 mm in diameter) in Hank's balanced salt solution. Each tumor piece was weighed and adjusted to be 150 mg. Mice were anesthetized with 4.3% trichloraldehyde hydrate. An incision was made through the left upper abdominal pararectal line. Then, peritoneal cavity was carefully exposed, and a part of serosal membrane in the middle of the greater curvature of stomach was mechanically injured using scissors. A tumor piece of 150 mg was fixed on each injured site of the serosal surface. The stomach was returned to the peritoneal cavity, and the abdominal wall and skin were closed. After metastatic models were made, the mice were randomly divided into N-desulfated heparin group (*n* = 10) and normal saline group (*n* = 10). One week after operation, the mice in N-desulfated heparin group received i.v. injections of N-desulfated heparin (Shanghai Institute of Cell Biology, Chinese Academy of Sciences, 10 mg/kg·d) twice weekly for 3 weeks. The mice in normal saline group received i.v. injections of normal saline (100 *μ*L) twice weekly for 3 weeks. The mice were weighed twice weekly.

### 2.5. Sample Collection and Pathological Examination

All animals were sacrificed at week 6 after implantation. An incision was made through the abdominal wall, and then peritoneal cavity was carefully exposed. Tumors growing on the stomach wall were removed and fixed in 10% formalin and processed for routine paraffin embedding. Tissues from all organs and lymph nodes were collected and fixed in 10% formalin and processed for routine paraffin embedding after careful macroscopic examination. Four-micron-thick sections were stained with hematoxylin and eosin and evaluated histologically for liver metastasis or lymph node metastasis or other organ metastasis under microscope.

### 2.6. Mean Microvascular Density of Tumor (MVD)

Immunostaining was performed using a labeled streptavidin biotin method. Four-micron-thick sections were deparaffinized in xylene and rehydrated with graded alcohol. Immunohistochemical staining was carried out to detect CD34 expression following the manufacturer's protocol. The concentration of anti-CD34 antibody was 1 : 300. MVD (CD34-positive microvessels) was calculated under 200-fold microscope. The modified Weidner's method was used for the evaluation of MVD according to CD34 endothelial cell immunostaining. For the microvessel counting, positive stainings for MVD in 5 most highly vascularized areas in each section were counted in 200 × fields. MVD was expressed as average of the microvessel count in the areas.

### 2.7. Detection of bFGF Expression

Immunostaining was performed using a labeled streptavidin biotin method. Four-micron-thick sections were deparaffinized in xylene and rehydrated with graded alcohol. Immunohistochemical staining was carried out to detect bFGF expression following the manufacturer's protocol. The concentration of anti-bFGF antibody was 1 : 60. Positive cells under 10% were defined as positive +, over 10% as positive ++. Positive expression was defined as positive + or positive ++.

### 2.8. Detection of bFGF mRNA Expression

bFGF primers and probe used are bFGF f: 5′-GTCACGGAAATACTCCAGTTG, bFGF r: 5′- CCGTTTTGGATCCGAGTTTATACT-3, bFGF probe: 5′-TGTGGCACTGAAACGAACTGGG-3. bFGF mRNA was isolated by method of Trizol. Synthesis of the first strand cDNA was performed according to the instructions delivered with reverse transcription kit, using human bFGF antisense strand primers and reverse transcriptase. After 1 h incubation at 37°C, samples were heat inactivated for 3 min at 95°C and kept at −80°C until use. Aliquots of 5 *μ*L of cDNA were amplified in a final volume of 50 *μ*L using PCR buffer at the presence of 1 *μ*L of Taq DNA polymerase and 0.5 *μ*L of bFGF probe. Samples were amplified at 93°C for 2 min, at 93°C for 0.5 min, and at 55°C for 1 min followed by 40 cycles. Real-time PCR was carried out in an automated real-time PCR cycler (American ABI 7000).

### 2.9. Statistical Analysis

All data were expressed as mean ± SD. Student's  *t*-test and *χ* ^2^ precise method were used to determine changes in different groups. *P* < 0.05 was considered statistically significant.

## 3. Results

### 3.1. Inhibition of N-Desulfated Heparin on Metastasis of Human Gastric Cancer

All mice developed localized tumors at the implanted site, which were poorly differentiated adenocarcinomas under microscope. Tumor growth did not differ significantly between the animals treated with normal saline or with N-desulfated heparin. Of the 10 animals treated with normal saline, 9 developed metastatic tumors in regional lymph nodes, 8 in liver, and 6 in other organs. However, after the mice were treated with N-desulfated heparin for 3 week, metastasis of tumor was inhibited significantly. Of the 10 animals treated with N-desulfated heparin, 2 developed metastatic tumors in liver. The metastatic rate was higher in mice treated with normal saline than in those treated with N-desulfated heparin (90% versus 20%, *P* < 0.05). N-desulfated heparin had no significant effect on body changes in NOD SCID mice. No bleeding complications were found in N-desulfated heparin group.

### 3.2. Effect of N-Desulfated Heparin on MVD

In normal saline-treated mice, many CD34 positively stained vessels were diffusely located and formed tube-like structures in tumor. However, they were almost absent in N-desulfated heparin-treated mice. The MVD was significantly lower in N-desulfated heparin-treated mice than in normal saline-treated mice (4.7 ± 1.8 versus 9.1 ± 3.4,  *t* = 3.617, *P* < 0.05).

### 3.3. Effect of N-Desulfated Heparin on bFGF Protein Expression

Under microscope, bFGF positive immunostaining was found in cytoplasm of cancer cells. The rate of bFGF positive expression was higher in normal saline group than in N-desulfated heparin group (*P* < 0.05, Table 1).

### 3.4. Effect of N-Desulfated Heparin on bFGF mRNA Expression in Gastric Tissue of NOD SCID Mice

bFGF mRNA expression in gastric tissue of NOD-SCID mice detected by real-time PCR was higher in normal saline group than in N-desulfated heparin group (ct value 19.51 ± 1.01 versus 22.55 ± 1.36, *P* < 0.05). 

### 3.5. Effects of N-Desulfated Heparin on bFGF Protein Expression In Vitro

The bFGF expression of human gastric cells was significantly increased with the extension of time in vitro (12 h versus 24 h, *P* < 0.05). With 0.1 mg/mL or 1 mg/mL N-desulfated heparin for 12 h and 24 h, bFGF expression was decreased significantly compared with control group (Table 2, *P* < 0.05). In 0.1 mg/mL and 1 mg/mL N-desulfated heparin groups, bFGF expression was decreased significantly at 24 h than at 12 h (*P* < 0.05). The results indicated that the inhibitory effect of N-desulfated heparin on bFGF expression of human gastric carcinoma cells was dose and time dependent.

### 3.6. Effect of N-Desulfated Heparin on bFGF mRNA Expression of Gastric Cancer SGC-7901 Cells

**Figure 1:**
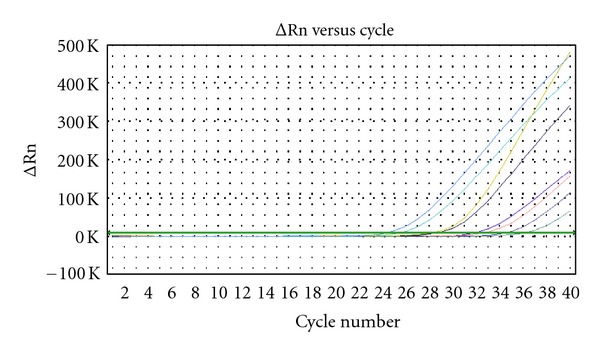
bFGF mRNA expression of human gastric cancer SGC-7901 cell in 0.1 mg/mL, 1 mg/mL N-desulfated heparin groups, and control group.

In each of N-desulfated heparin groups, bFGF mRNA expression was decreased compared with control group (*P* < 0.05). The higher ct values mean the lower concentration of bFGF mRNA. The inhibitory effect of N-desulfated heparin on bFGF mRNA expression of human gastric carcinoma cells in vitro was associated with doses.  Table 3 showed that the ct values in 0.1 mg/mL N-desulfated heparin groups were higher than in control group (*P* < 0.05) and that the ct values were higher in 1 mg/mL N-desulfated heparin group compared with 0.1 mg/mL N-desulfated heparin group (*P* < 0.05), suggesting that it had dose-dependent effects. In the same N-desulfated heparin concentration, the expression of bFGF mRNA at 24 h was lower than at 12 h (*P* < 0.05, 1 mg/mL N-desulfated heparin group), suggesting that the inhibition of N-desulfated heparin on bFGF mRNA expression of human gastric carcinoma cells had time-dependent effects (Figure 1, Table 3, *P* < 0.05).

## 4. Discussion

Tumor invasion and metastasis is a multistep process that promotes the spread of the cancer from primary sites to distant locations. Recent studies have showed that angiogenesis is a critical determinant of solid tumor metastasis, and antiangiogenic therapy plays an important role in improving prognosis of patients with gastric carcinoma [15–17]. VEGF represents a target for antiangiogenic therapies in a wide spectrum of diseases, including cancer. As a novel strategy to generate nonanticoagulant antiangiogenic substances exploiting binding to VEGF while preventing receptor engagement, Pisano et al. [18] assessed the VEGF-antagonist activity of a low-molecular-weight (LMW) compound generated by depolymerization of an undersulfated glycol-split heparin derivative. Unlike heparin, it was unable to present 125I-VEGF165 to its high-affinity receptors in endothelial cells and inhibited VEGF165-induced neovascularization in the chick embryo chorioallantoic membrane. Therefore, undersulfated, LMW glycol-split heparins may provide the basis for the design of novel nonanticoagulant angiostatic compounds. In addition, it has been reported that undersulfated and glycol-split heparins have endowed with antiangiogenic activity. Heparin binds to bFGF and promotes the formation of ternary complexes with endothelial cell surface receptors, inducing an angiogenic response [19]. N-desulfated heparin, a modified heparin, is known to have more significantly reduced anticoagulant activity (1/76 of heparin) than O-desulfated heparin (5%–30% of heparin) or N-acetylated heparin (10% of heparin) [20]. However, there is no report so far on the effect of N-desulfated heparin on tumor metastasis. Therefore, the effect of N-desulfated heparin on tumor metastasis, angiogenesis, and bFGF expression was observed in mouse model of orthotopic implantation of human gastric carcinoma tissue.

In the present study, tumor metastasis was inhibited significantly by N-desulfated heparin. To evaluate the effect of N-desulfated heparin on angiogenesis, immunohistochemical staining of CD34 in tumors was carried out. The results showed that N-desulfated heparin significantly inhibited angiogenesis in these tumors. Lee et al. [21] generated LHD or orally active heparin using low-molecular-weight heparin (LMWH) and deoxycholic acid that could be effectively absorbed in the gastrointestinal tract, making it an attractive candidate as an oral drug for antiangiogenic cancer therapy. Heparin oligosaccharides may be an inhibitor of the biological activity of bFGF on Caco-2 cells [22]. Norrby [23] found that 2.5 kDa and 5.0 kDa heparin fragments could specifically inhibit microvessel sprouting and network formation in VEGF165-mediated mammalian angiogenesis. Ono et al. [24] demonstrated that periodate-treated, nonanticoagulant heparin-carrying polystyrene (NAC-HCPS) affected angiogenesis and inhibited subcutaneous-induced tumour growth and metastasis to the lung. Mousa and Mohamed [25] have demonstrated antiangiogenic activity of the low-molecular-weight heparin, tinzaparin. Naggi et al. [13] has reported that N-acetylated and glycol-split heparins are potential antiangiogenic and antimetastatic agents which are more effective than unmodified heparin, suggesting that N-desulfated heparin can inhibit tumor metastasis by inhibiting angiogenesis.

bFGF is a ubiquitously expressed polypeptide growth factor that is normally sequestered in the extracellular matrix of healthy tissues [26]. It is also expressed by many human cancer cells, including prostate carcinoma and melanoma cells and is believed to be important for the formation of tumor vasculature [27, 28].

In this study, the rate of bFGF positive expression was higher in normal saline group than in N-desulfated heparin group and bFGF mRNA expression was higher in normal saline group than in N-desulfated heparin group, demonstrating that N-desulfated heparin can significantly inhibit the bFGF expression of cancer cells. In vitro, the bFGF expression of human gastric cells was significantly increased with the extension of time. Treated with 0.1 or 1 mg/mL N-desulfated heparin for 12 and 24 h, bFGF expression was decreased significantly. Moreover, in each of N-desulfated heparin groups, bFGF mRNA expression was decreased compared with control group. bFGF mRNA expression was lower in 1 mg/mL N-desulfated heparin group than 0.1 mg/mL N-desulfated heparin group. In the same N-desulfated heparin concentration, the expression of bFGF mRNA at 24 h was lower than that at 12 h. Therefore, the inhibition of N-desulfated heparin on bFGF mRNA expression of human gastric carcinoma cells had dose- and time-dependent effects. The results suggest that N-desulfated heparin inhibits tumor angiogenesis by inhibiting expression of bFGF.

Sartippour et al. [29] concluded that nipple fluid bFGF levels were progressively elevated in high-risk and cancerous breasts compared with benign breasts. Barclay et al. [30] found that overexpression of bFGF mRNA by comparison with tumors underexpressing bFGF was associated with significantly increased risk for tumor recurrence. Hatziapostolou et al. [31] has showed that bFGF is a pleiotropic growth factor that has been implicated in prostate cancer formation and progression. According to the study, they found that exogenous bFGF significantly increased human prostate cancer LNCaP cell proliferation and migration. Heparin affin regulatory peptide (HARP) or pleiotrophin seems to be an important mediator of bFGF stimulatory effects. bFGF, through FGF receptors (FGFRs), significantly induced HARP expression and secretion by LNCaP cells and increased luciferase activity of a reporter gene vector carrying the full-length promoter of HARP gene. Activation of FGFR by bFGF in LNCaP cells leads to NAD(P)H oxidase-dependent hydrogen peroxide production, phosphorylation of ERK1/2 and p38, activation of AP-1, increased expression and secretion of HARP, and, finally, increased cell proliferation and migration. Pardo et al. [32] demonstrated that bFGF increased the expression of antiapoptotic proteins, XIAP, and Bcl-X(L) and triggered chemoresistance in SCLC cells. They found that these effects were mediated through the formation of a specific multiprotein complex comprising B-Raf, PKCepsilon, and S6K2. In a tetracycline-inducible system, increased S6K2 kinase activity triggers upregulation of XIAP, Bcl-X(L) and prosurvival effects. Zhao et al. [33] has reported that the measurement of plasma levels of such angiogenic factors as VEGF, bFGF, and MMP-9 in advanced NSCLC is helpful for prediction of metastasis tendency and evaluation of prognosis. Expression of the mRNAs of VEGF, flt-1, flk-1, and flg-1 (a receptor for bFGF) was analyzed by reverse transcriptase polymerase chain reaction (RT-PCR) and in situ hybridization (ISH) with cRNA probes. VEGF, bFGF, flt-1, and flk-1 were immunohistochemically detected in the neoplastic cells in HSAs; the staining intensity was stronger in HSAs than in hemangiomas [34].

In the present study, hemorrhage was never observed in N-desulfated heparin treated mice, suggesting that N-desulfated heparin has no obvious anticoagulant activity. In conclusion, bFGF produced by cancer cells is an angiogenic factor in human cancer tissue and plays an important role in tumor metastasis. N-desulfated heparin inhibits tumor metastasis by inhibiting expression of bFGF and angiogenesis. N-desulfated heparin can be used in the treatment of tumor metastasis.

## Figures and Tables

**Table 1:** Effect of N-desulfated heparin on bFGF expression in gastric cancer in vivo (mean ± SD).

Groups	*n*	−	+	++	Positive rate (%)
Normal saline group	10	1	2	7	90
N-desulfated heparin group	10	8	1	1	20

N-desulfated heparin group versus normal saline group, *P* < 0.05.

**Table 2:** bFGF expression of gastric cancer SGC-7901 cells in 0.1 mg/mL, 1 mg/mL N-desulfated heparin groups, and control group (pg/mL, $\bar{x}\pm s$, *n* = 3).

Groups	12 hr	24 hr
Control group	17.724 ± 0.173	19.690 ± 0.111*
0.1 mg/mL N-desulfated heparin group	15.313 ± 0.394^∆^	13.301 ± 0.358^∆∗^
1 mg/mL N-desulfated heparin group	12.173 ± 0.063^∆#^	11.174 ± 0.286^∆∗#^

*24 hr versus 12 hr, *P* < 0.05; ^∆^different concentrations (0.1 mg/mL, 1 mg/mL) of N-desulfated heparin versus control group, *P* < 0.05; ^#^1 mg/mL N-desulfated heparin group versus 0.1 mg/mL N-desulfated heparin group, *P* < 0.05.

**Table 3:** bFGF mRNA expression of gastric cancer SGC-7901 cell in different concentrations (0.1 mg/mL, 1 mg/mL) of N-desulfated heparin groups and control group (ct value, $\bar{x}\pm s$, *n* = 3).

	12 hr	24 hr
Control group	25.956 ± 0.505	24.490 ± 0.145*
0.1 mg/mL N-desulfated heparin group	30.923 ± 0.612^∆^	32.493 ± 0.358^∆^
1 mg/mL N-desulfated heparin group	33.826 ± 0.349^∆#^	35.446 ± 0.299^∆∗#∗^

*24 hr versus 12 hr, *P* < 0.05; ^∆^different concentrations (0.1 mg/mL, 1 mg/mL) of N-desulfated heparin versus control group, *P* < 0.05; ^#^1 mg/mL N-desulfated heparin group versus 0.1 mg/mL N-desulfated heparin group, *P* < 0.05.
